# Assessing the performance of an automated breast treatment planning software

**DOI:** 10.1002/acm2.13228

**Published:** 2021-03-25

**Authors:** Irena Dragojević, Jeremy D. P. Hoisak, Gina J. Mansy, Douglas A. Rahn, Ryan P. Manger

**Affiliations:** ^1^ Department of Radiation Medicine and Applied Sciences University of California – San Diego 3855 Health Sciences Dr. La Jolla CA 92037 USA

**Keywords:** automation, automated planning, breast cancer, breast radiotherapy, dosimetry, treatment planning

## Abstract

**Purpose:**

To assess the dosimetric performance of an automated breast planning software.

**Methods:**

We retrospectively reviewed 15 breast cancer patients treated with tangent fields according to the RTOG 1005 protocol and 30 patients treated off‐protocol. Planning with electronic compensators (eComps) via manual, iterative fluence editing was compared to an automated planning program called EZFluence (EZF) (Radformation, Inc.). We compared the minimum dose received by 95% of the volume (D95%), D90%, the volume receiving at least 105% of prescription (V105%), V95%, the conformity index of the V95% and PTV volumes (CI95%), and total monitor units (MUs). The PTV_Eval structure generated by EZF was compared to the RTOG 1005 breast PTV_Eval structure.

**Results:**

The average D95% was significantly greater for the EZF plans, 95.0%, vs. the original plans 93.2% (*P* = 0.022). CI95% was less for the EZF plans, 1.18, than the original plans, 1.48 (*P* = 0.09). D90% was only slightly greater for EZF, averaging at 98.3% for EZF plans and 97.3% for the original plans (*P* = 0.0483). V105% (cc) was, on average, 27.8cc less in the EZF breast plans, which was significantly less than for those manually planned. The average number of MUs for the EZF plans, 453, was significantly less than original protocol plans, 500 (*P* = 8 × 10^−6^). The average difference between the protocol PTV volume and the EZF PTV volume was 196 cc, with all but two cases having a larger EZF PTV volume (*P* = 0.020).

**Conclusion:**

EZF improved dose homogeneity, coverage, and MU efficiency vs. manually produced eComp plans. The EZF‐generated PTV eval is based on the volume encompassed by the tangents, and is not appropriate for dosimetric comparison to constraints for RTOG 1005 PTV eval. EZF produced dosimetrically similar or superior plans to manual, iteratively derived plans and may also offer time and efficiency benefits.

## 1 INTRODUCTION

Breast cancer is the most common type of cancer and there are ~ 300,000 patients annually diagnosed in the United States.^1^, ^2^ Adjuvant whole breast radiotherapy is often used following surgery for stage I‐III breast cancer as part of breast conservation therapy, and breast radiation may be used in oligometastatic breast cancers as well. With an aging population as well as increasing therapeutic use of radiation for all sites, the number of patients being treated is expected to increase,^3^ challenging available treatment planning and delivery resources. Recent advances such as multi‐criteria optimization^4^ and knowledge‐based planning^5^ have reduced the time and planner effort required to generate high‐quality intensity‐modulated radiation therapy (IMRT) plans for sites such as the head & neck, lung, prostate, and brain. However, these advances have not translated into faster or more efficient radiation treatment planning for breast, as IMRT is not usually required for most breast cancer patients receiving radiation.^6^ Whole breast and chest wall radiation therapy using 3D‐CRT is typically planned with a forward‐planned field‐in‐field (FiF) or electronic compensator (eComp) technique that modulates the radiation fluence to achieve adequate dose homogeneity in the target tissue. The conventional breast 3D‐CRT treatment planning process at our institution includes the following steps: 1. Patient undergoes computed tomography (CT) simulation scan. 2. Planner imports the simulation CT data set; 3. Physician sets field angles and borders; 4. Planner performs fluence and dose optimization; 5. Physician reviews plan and either requests dosimetric changes or approves the plan; 6. Physicist reviews plan, performs quality assurance checks, and either requests changes or approves for treatment. A manual, iterative approach requires the full attention of the planner (e.g., they cannot multitask by running an optimization task in the background while working on another treatment plan). Seeking physician input and review for each dose optimization step can be time‐consuming and involves several handoffs between clinic staff. For each iterative step requiring physician plan review they must re‐familiarize themselves with the patient’s case and planning requirements, increasing the chances of error. In addition, in clinics where physicians may only be in the clinic a few days of the week the entire planning process may be delayed even further if multiple reviews are required. This iterative fluence and dose optimization followed by physician plan review is currently the most time‐consuming step of whole breast radiotherapy planning. Therefore, automating fluence generating step can significantly expedite the treatment planning process and overall treatment plan quality. A software tool that recently became commercially available, EZFluence (EZF) version 1.4 (Radformation, Inc.), automates optimal fluence generation. The goal of this work was to assess the performance of EZF automated breast treatment planning software and compare it to manual planning techniques based on a variety of dosimetric plan quality indices.

## 2 METHODS

### 2.A Treatment planning software

EZF is software that functions as a plug‐in script for the Eclipse Treatment Planning System (TPS), (Varian Medical Systems, Palo Alto, CA). Accessing the Eclipse application programming interface (API), it communicates with the Varian database, and generates an initial optimal fluence. Generated fluence is based on midpoint and hot spot criteria selected by the user, who is then able to select the best fluence among several options. It is possible for the user to further manually edit this fluence in EZF or in the Eclipse TPS after it is exported from EZF.

### 2.B Selection criteria

To effectively assess the effectiveness of the EZF algorithm, it must be compared to known radiation treatment planning benchmarks, such as those described in the radiation therapy section of RTOG 1005 protocol (a phase III trial of accelerated whole breast irradiation with hypofractionation plus concurrent boost versus standard whole breast irradiation plus sequential boost for early stage breast cancer).^7^

The selection criteria for this study include patients who (a) received radiation treatment under RTOG 1005 protocol or b) were treated according to the protocol guidelines but were never formally enrolled in the trial. Patients treated with regional nodal radiotherapy using IMRT or RapidArc volumetric‐modulated arc therapy (VMAT) were excluded.

In addition, due to the relatively low number of patients who were enrolled in or treated according to the protocol, we also include non‐protocol cases, to assess plan quality and planning efficiency. Selection criteria for these non‐protocol cases were a) breast tangent plans with fluence editing or b) chest wall tangent plans with fluence editing technique. Tangent eComp plans calculated on deep inspiration breath hold (DIBH) scans were also included in the study. This study was approved by the University of California San Diego Internal Review Board.

### 2.C Automated breast planning

The eComp breast tangent plans that were manually planned in Eclipse treatment planning software according to RTOG 1005 were replanned using EZF automated breast planning software. Using the same field borders, EZF was used to automatically derive fluences for the eComp. EZF plans were normalized such that the maximum point dose was less than or equal to the protocol plan. EZF software predicts the maximum point dose, and if the point dose is less than the original plan, it was unchanged. If the maximum point dose in EZF plan was lower than the original plan, it was not normalized higher because this results in an over‐normalized, dosimetrically worse plan. Occasionally, the un‐normalized EZF maximum point dose exceeded the original plan, and in that case, the plan was normalized to match the maximum point dose in the original, manual plan. This automated replanning process was then used for the non‐protocol plans.

### 2.D Plan evaluation

The following dosimetric parameters were compared: the minimum dose received by 95% of the volume (D95%), D90%, the volume receiving at least 105% of prescription (V105%), V95%, CI95% (the quotient of V95 and PTV volume), and total monitor units (MU). D95 and D90 were evaluated on physician‐contoured PTV_Eval when available (protocol plans), and PTV_Eval_EZ that is generated by EZF was used in non‐protocol patients, as that was the only one available. EZF auto‐contours a planning structure called PTV_Eval_EZ by cropping the irradiated volume 5 mm from the field edge, the skin, and specified organs at risk (OAR) (e.g., lung, heart, etc). EZF uses this structure as an optimization structure. This PTV_Eval_EZ was compared against the manually contoured RTOG 1005 whole breast PTV_Eval structure according to volume differences and the Dice similarity coefficient. Since non‐protocol patients did not have manually contoured PTV_Eval, this comparison was only performed for the 15 protocol patients. Mean heart dose and ipsilateral lung volume receiving 20 Gy (V20) were also compared to determine if automated planning affected OAR sparing.

### 2.E Timing study

A timing study was devised to estimate the time saved by introducing automated EZF software into the workflow. Board‐certified medical dosimetrists were tasked with creating manual and automated treatment plans. They self‐reported the time required to generate automated plans. The conventional breast tangent treatment planning process at our institution included the following steps: 1. Planner imports the simulation CT data set; 2. Physician sets field angles and borders; 3. Planner performs dose optimization; 4. Physician reviews plan and either requests dosimetric changes or approves the plan; 5. Physicist reviews plan and either requests changes or approves for treatment. In the new treatment planning process, after the physician sets field angles and borders, EZF generates an optimal fluence and the planner and physician choose from a set of plans with similar coverage or hot spots while providing metrics such as V105, max point dose, and D95 in real time. A timing study of 45 patients was conducted to compare the average times to generate a clinically acceptable plan using the conventional and automated planning workflows.

### 2.F Statistical analysis

Data from the manual plans were compared to the automated plans using the two‐tailed paired *t* test with an alpha of 0.05. Due to the small sample size for protocol‐only patients, data from the complete data set, both protocol and non‐protocol, were used for evaluation. The only exception is CI95%, which was only evaluated for the protocol patients, because manually contoured PTV_Eval structures were not available for non‐protocol patients.

## 3 RESULTS

**Fig. 1:**
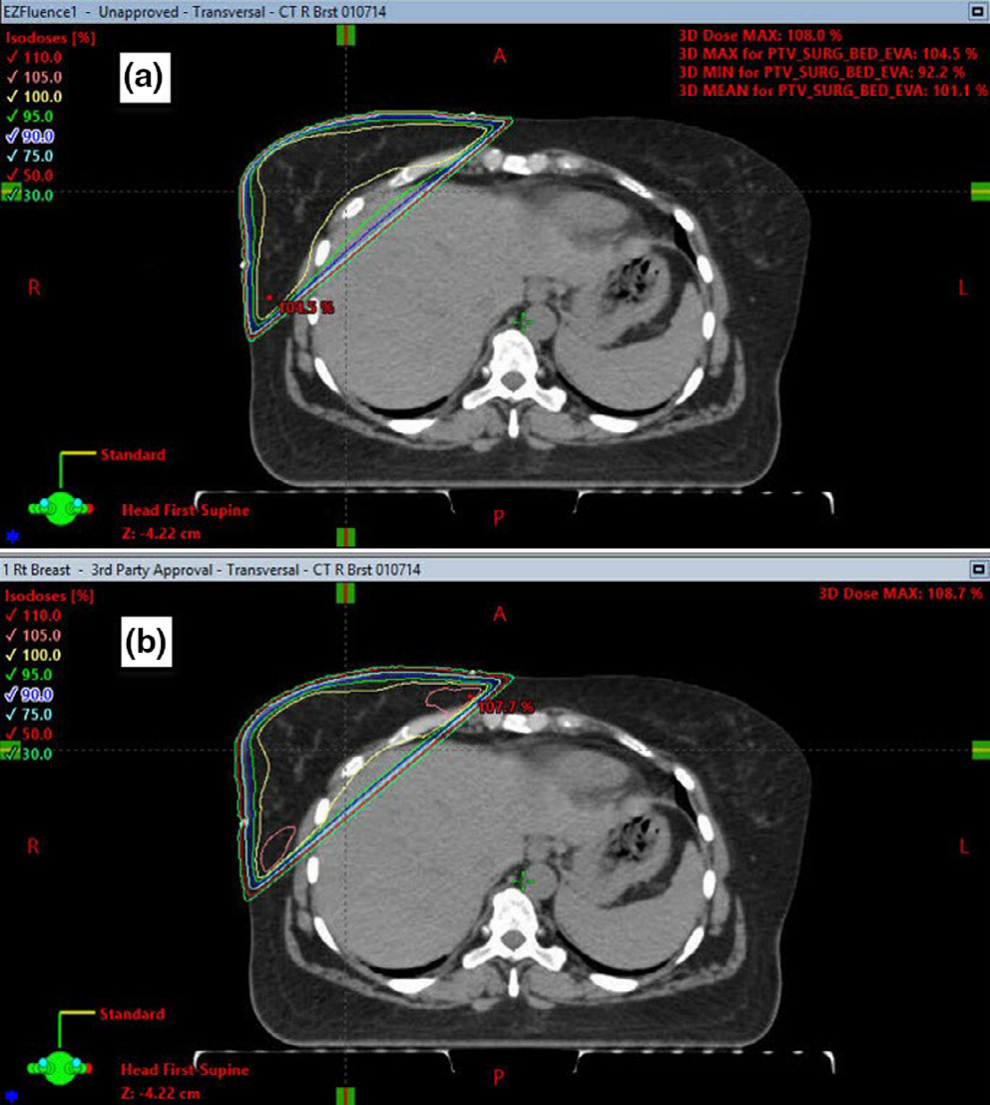
Representative plan comparison between the EZF plan a) and RTOG 1005 manually planned protocol plan b), showing dose distributions in the axial plane.

Fifteen breast patients planned according to RTOG 1005 protocol and 30 non‐protocol breast patients were identified. The 15 protocol plans consisted of six left breast plans and eight right breast plans, and one right chest wall. The 30 non‐protocol plans consisted of 10 left breast, 10 right breast, five right chest wall, and five left chest wall patients. All were originally planned using a manual iterative planning method and treated with an eComp technique. They were then replanned with the EZF tool and compared (Fig. 1).

**Fig. 2:**
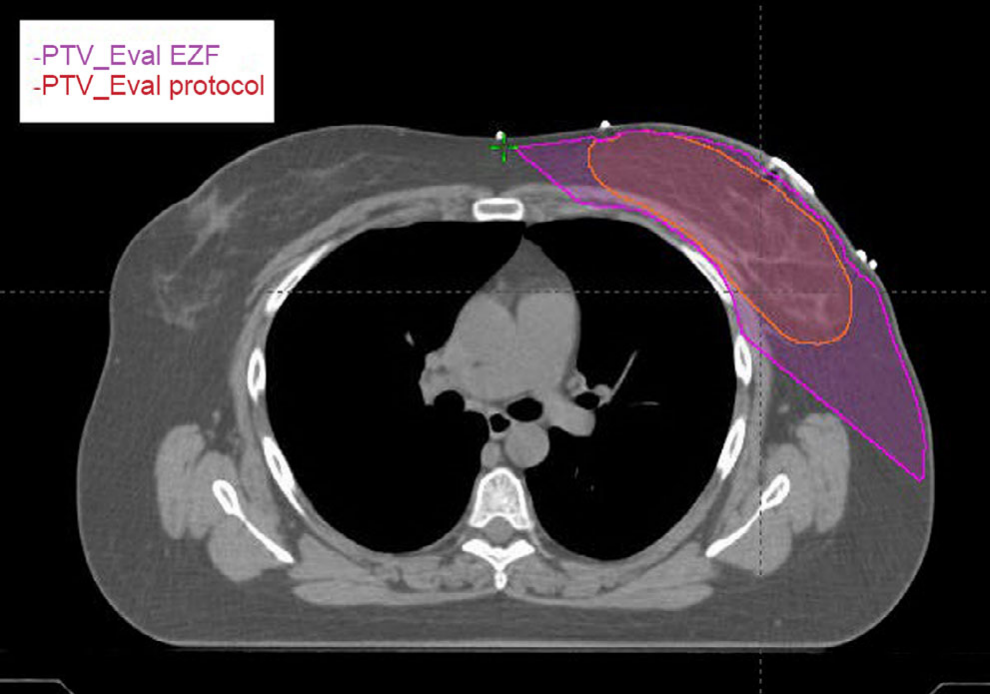
Shown is a typical PTV_Eval contoured by a physician and auto‐contoured PTV_Eval_EZ by EZF.

When comparing all 45 analyzed plans, the V105% (cc) hot spot, was, on average, 27.8 cc less in the EZF breast plans (*P* = 0.026). Compared to the manual plans, the EZF average D95% target coverage was significantly greater (95% vs. 93.2% *P* = 0.022). The average number of monitor units (MU) for the EZF plans was 453, significantly less than for the manual protocol plans where average MU was 500 (*P*<<0.01). The RTOG 1005 protocol data subset had manually contoured PTV_Eval structures available, and CI95% was calculated for both manual and EZF plans. For the manual plans, CI95% was 1.48, while it was more conformal for the EZF plans, CI95%=1.18 (*P* = 0.095). The EZF PTV_Eval tended to be larger than those contoured by physicians. EZF auto‐contours the PTV by including any tissue within the field minus 5 mm from the skin, field borders, and OAR (Fig. 2). EZF overestimates breast tissue because it does not differentiate between breast and non‐breast tissue, and also because the definition of PTV_Eval in RTOG 1005 is slightly different (RTOG 1005 PTV_Eval contours are defined as tumor bed volume with 1.7 cm margin which is then cropped by 5mm from the skin and chest wall). The average Dice similarity coefficient between the manually contoured PTV_Eval and PTV_Eval_EZ was 0.79 (range 0.46–0.92).

Mean heart doses and lung V20 volumes were compared to determine if the automated plans and manual plans had significant differences. However, the differences were shown to be statistically insignificant, with p‐values ranging from 0.88 to 1. The results are shown in Table 1.

**Table 1:** The mean, standard deviations, and *P*‐values for mean heart dose and lung V20 evaluations.

	Metric	Manual plan (Mean ± SD)	EZF plan (Mean ± SD)	*P*‐value
Protocol plans vs. EZF	Mean heart dose (Gy)	0.62 (±0.43)	0.64 (±0.47)	0.88
	Lung V20 (%)	16.6 (±29.42)	17.10 (±31.27)	0.93
Non‐protocol plans vs. EZF	Mean heart dose (Gy)	0.72 (±0.49)	0.71 (±0.47)	0.92
	Lung V20 (%)	11.57 (±6.81)	11.55 (±6.48)	0.99
Total data vs. EZF	Mean heart dose (Gy)	0.69 (±0.47)	0.69 (±0.46)	1.00
	Lung V20 (%)	13.04 (±17.30)	13.33(±18.24)	0. 94

Overall, EZF produced eComp plans with improved dosimetric homogeneity, coverage, and MU efficiency than the manually edited plans. Statistics for both protocol and non‐protocol data sets are shown in Table 2, along with the combined data set. Due to the low number of protocol data points, the differences between the manual and automated plans for this data set on its own were statistically not significant.

**Table 2:** The mean, standard deviations, and *P*‐values for plan quality evaluation parameters.

	Metric	Manual plan (Mean ± SD)	EZF plan (Mean ± SD)	*P*‐value
Protocol plans vs. EZF	V105% (cc)	128.0 (±175)	73.0 (±77)	0.111
	V105/V95 (%)	77.4 (±8.35)	15.8 (±3.59)	0.0897
	D95 (%)	87.8 (±18.7)	91.8 (±11.9)	0.0707
	D90 (%)	96.6 (±5.4))	98.4 (±2.32)	0.133
	MU	495 (±102)	457 (±33.8)	0.175
	CI95%	1.48 (±0.58)	1.18 (±0.13)	0.0945
Non‐protocol plans vs. EZF	V105% (cc)	81.2 (±71.1)	68.7 (±67.5)	0.102
	V105/V95 (%)	7.27 (±5.44)	6.05 (±4.84)	0.0695
	D95 (%)	95.7 (±3.8)	96.5 (±2.6)	0.146
	D90 (%)	97.5 (±3.3)	98.2 (±1.7)	0.192
	MU	501 (±84)	447.7 (±83)	1.75E‐11
Total data vs. EZF	V105% (cc)	94.4 (±118)	66.5 (±69.3)	0.0257
	V105/V95 (%)	7.2 (±6.5)	5.2 (±4.2)	0.00870
	D95 (%)	93.2 (±7.5)	95 (±11.9)	0.0219
	D90 (%)	97.3 (±4.2)	98.3 (±1.94)	0.0483
	MU	500 (±92)	453 (±71)	8.26E‐06

The top shows the RTOG 1005 protocol data compared to EZF, the middle is the non‐protocol data, and on the bottom is the combined data from both data sets. For D95 and D90 calculations, manually contoured PTV_Eval was used for the protocol plans, and PTV_Eval_EZ for the remaining plans where physician‐contoured PTV_Eval was not available. V105 and V95 were calculated based on the whole body contour.

This study also evaluated the average time required to generate a clinically acceptable plan using the conventional and automated EZF planning workflows. The conventional treatment planning process took 4–5 days on average from CT import to physician plan approval. The new automated method of creating and choosing a clinically acceptable plan took an average of 6 min, not including the manual setup of the fields. Time required to do the same plans manually ranged between 1 and 3 h, depending on the case complexity.

## 4 DISCUSSION

Breast cancer patients often constitute a significant portion of the total patient population treated in radiation oncology clinics. This comes as no surprise, since breast cancer is the most prevalent cancer and the second leading cause of cancer death among women.^1^ Breast cancer may comprise 30% or more of the patient volume in a typical radiotherapy clinic. Therefore, automating breast treatment planning has the potential to have significant impact on most radiation oncology clinics in terms of efficiency, plan quality, and time between simulation and treatment.

Manual fluence editing for breast treatment planning in Varian Eclipse TPS is a human‐driven, iterative process that is time‐consuming. Each iteration proceeds in a trial‐and‐error fashion, with the planner editing the fluence, calculating 3D dose, and evaluating the dose until “optimal” dosimetry is achieved. There are several published studies on automated treatment planning algorithms being implemented for various anatomical sites, including breast.^8^, ^9^, ^10^, ^11^, ^12^, ^13^, ^14^, ^15^, ^16^ Many institutions use in‐house developed software for plan automation, and while that is a feasible solution for those that already have it or have the resources to develop, maintain,and validate in‐house solutions, such an approach may not be accessible for many nonacademic clinics. Commercially available software such as EZF, therefore can provide an ideal solution for those that have limited time and resources.

Another benefit of automated treatment planning is a reduction in planner‐dependent, plan quality variability. However, the most significant benefit is in the time savings and improving the workflow efficiency. It can take 1‐3 h to manually create a breast treatment plan, depending on anatomy and level of experience of the planner. A timing study of four individual planners showed that on average, it took ~ 6 min to create an acceptable EZF plan, not including the time spent on setting up the fields and MLC blocks. This is comparable to what several others reported for other automated solutions, although included in their time estimate was automatic field setup as well.^10^, ^13^ Inability to automatically set the fields is the most significant shortcoming of EZF. Since the most time‐consuming step in manual breast treatment planning is fluence editing, this shortcoming does not significantly affect the workflow.

**Fig. 3:**
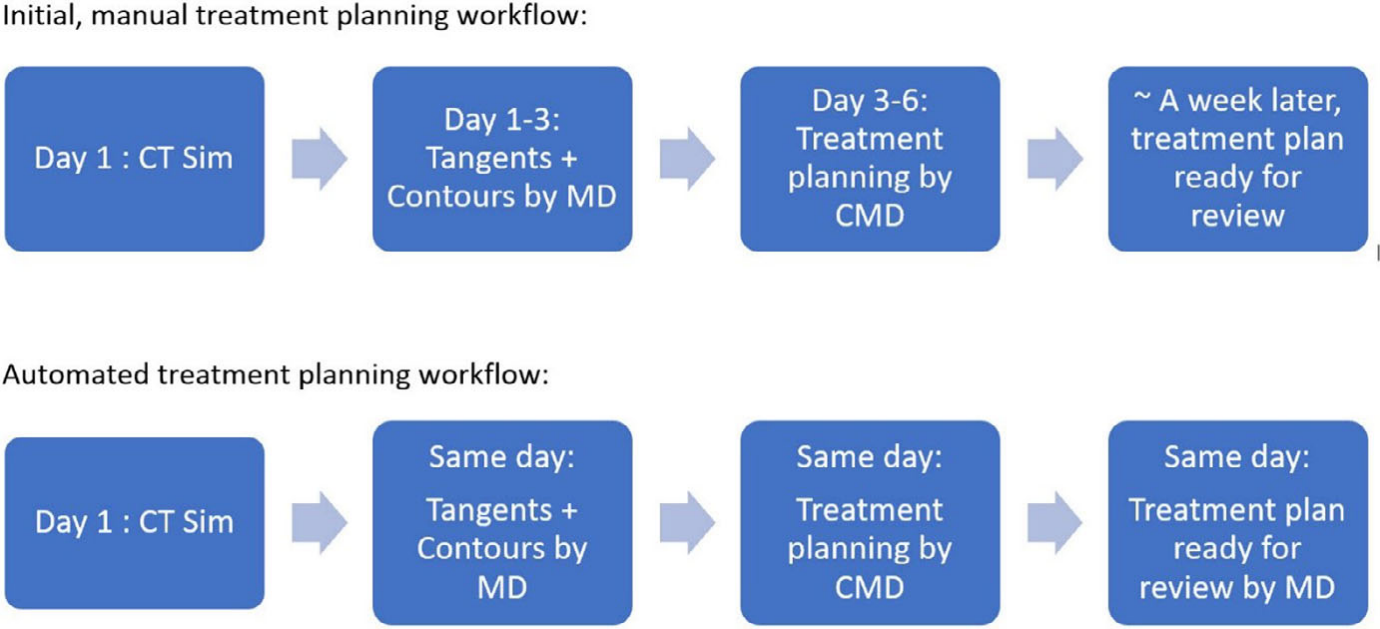
Workflow timeline with manual breast planning (top) and automated EZF planning (bottom).

In our clinic, time between simulation and treatment was shortened significantly. While manual fluence edited plans took a week between the initial simulation and treatment, EZF automated plans were completed in a day (Fig. 3). It was interesting to observe that the workflow improved even in areas that were not directly involved in treatment planning. For example, treatment approval often depended not only on a plan being completed but also on the physician schedule, which days they were in clinic and what time of the day they had scheduled for a given task. Shortening the time to plan increased the likelihood that a plan would get approved earlier in the day. In the old system, if a plan was not approved on a given day, it was likely that the physician would not review it until the next time they were in clinic, which could take a few days. Eliminating some of this waiting time has been most influential in shortening the turnaround time from simulation to treatment. Finally, EZF automation influenced how dosimetry approached planning. They became more likely to do the quick automated plans earlier in the day to get them out of the way rather than leaving them for a later time and running into the problem of physician not being there when the plan was ready for review. The development of an efficient breast planning workflow would be of tremendous benefit to clinics in underserved areas where staffing can be a challenge amid high patient loads. Same or next‐day breast planning would also be of benefit in remote or low‐ and middle‐income clinics where patients often travel great distances for treatment and would face an undue burden if simulation and treatment were widely spaced in time. While plan quality of automated eComp breast planning is the focus of this study, in future work, we plan to examine the efficiency of the EZF‐based breast planning process in more depth so that others can implement a similar planning strategy in their clinics.

## 5 CONCLUSION

EZF produces eComp plans with improved dosimetric homogeneity, coverage, and MU efficiency than manually edited plans. The EZF auto‐contoured PTV provides a consistent breast target to create dosimetrically similar or superior plans but is not equivalent to the RTOG 1005 PTV_Eval and should not be used for RTOG 1005 dosimetric evaluation.

The speed of planning and consistent plan quality are the strongest features of EZF. In this study, EZF significantly reduced the time and resources required to produce eComp breast plans at a significantly higher quality than manually planned cases. This allowed us to facilitate an accelerated, efficient treatment planning workflow.

## CONFLICT OF INTEREST

No conflict of interest.

## Author Contribution

All the authors participated in data analysis and manuscript composition.

## Data Availability

The data that support the findings of this study are available from the corresponding author upon reasonable request
